# Complete Genome Sequence of *Mesorhizobium sophorae* ICMP 19535^T^, a Highly Specific, Nitrogen-Fixing Symbiont of New Zealand Endemic *Sophora* spp.

**DOI:** 10.1128/genomeA.00958-17

**Published:** 2017-10-26

**Authors:** Sofie E. De Meyer, Dung Tuan Nguyen, Penghao Wang, Mitchell Andrews

**Affiliations:** a Centre for Rhizobium Studies, Murdoch University, Murdoch, Western Australia, Australia; b Laboratory of Microbiology, Department of Biochemistry and Microbiology, Ghent University, Ghent, Belgium; c Faculty of Agriculture and Life Sciences, Lincoln University, Lincoln, New Zealand

## Abstract

We report here the complete genome sequence of *Mesorhizobium sophorae* ICMP 19535^T^. This strain was isolated from *Sophora microphylla* root nodules and can nodulate and fix nitrogen with this host and also with *Sophora prostrata*, *Sophora longicarinata*, and *Clianthus puniceus*. The genome consists of 8.05 Mb.

## GENOME ANNOUNCEMENT

Most legume species (plant family *Leguminosae*) can fix atmospheric nitrogen via symbiotic bacteria (“rhizobia”) in root nodules, which can give them an advantage under low-nitrogen soil conditions (1, 2). New Zealand is geographically isolated, and this separation has given rise to a unique native flora (3). There are four genera of indigenous legumes on the main New Zealand islands, and all of them are in the subfamily *Papilionoideae* and all nodulate. These genera are *Carmichaelia*, *Clianthus*, and *Montigena* in the tribe Galegeae, and *Sophora* is within the tribe Sophoreae (4, 5).

Forty-eight rhizobial isolates from four New Zealand native *Sophora* spp. sampled at eight different field sites were separated into eight groups and three individual isolates on the basis of their concatenated *recA*, *glnII*, and *rpoB* gene sequences, while showing almost identical unique *nodA* and *nodC* gene sequences (6). Seven of these groups have been formally identified as new species, one of which is *Mesorhizobium sophorae* ICMP 19535^T^ (7, 8). This relationship between New Zealand native *Sophora* spp. and *Mesorhizobium* spp. with specific nodulation gene sequences is highly specific (9).

Genomic DNA was isolated using the GENTRA Pure-Gene kit (Qiagen). The genome sequence was determined using the PacBio RS II (Pacific Biosciences) platform at ChunLab, Inc. (South Korea). Raw sequences were assembled with PacBio SMRT Analysis version 2.0 software (Pacific Biosciences). Gene prediction was performed using tRNA-scan version 1.3.1 (10) for the tRNA search, Cmsearch (INFERNAL version 1.0.2) and Rfam version 12.0 for the rRNA and noncoding RNA searches, and gPilerCR version 1.06 (11) and CRT1.2 (12) for the clustered regularly interspaced short palindromic repeat (CRISPR) searches. Functional annotation was performed by a homology search against the KEGG (13), SEED (14), Swissprot (15), and eggNOG (16) databases. In total, 8,049,106 bp of sequence information was obtained with a total of 24 contigs. The average contig size was 334,479 bp, with the largest being 1,626,386 bp and the shortest 14,820 bp. The genome features an average GC content of 62.22%, and the *N*_50_ value was 666,711 bp. A total of 7,659 protein-coding sequences and 6 rRNAs and 58 tRNAs were predicted.

The genomic sequence comparison against other sequenced *Mesorhizobium* strains indicated that around 70% of the sequences of ICMP 19535^T^ are highly conserved. However, ICMP 19535^T^ demonstrated segments of unique sequences and genomics rearrangements. The closest strains that were matched to ICMP 19535^T^ include the rhizobia *Mesorhizobium ciceri* bv.* biserrulae* WSM1284 and *Mesorhizobium loti* NZP2037. Preliminary sequence analysis revealed that the ICMP 19535^T^ genome contains many key genes known to be associated with legume symbiosis. A number of clusters of nodulation genes (*nod*) were identified, and, interestingly, one cluster of *nod* genes is located on a genomic island that contains multiple *nif* genes, which are known to encode enzymes involved in nitrogen fixation. Moreover, a cluster of *fix* genes was discovered. Further analysis of the genome will lead to a better understanding of the strain’s genomic mechanisms that regulate nodulation and nitrogen fixation associated with native New Zealand legume symbioses.

### Accession number(s).

This whole-genome shotgun project has been deposited at DDBJ/ENA/GenBank under accession number NNRI00000000. The version described here is the first version, NNRI01000000.
